# Enhanced anti−tumor efficacy of “IL−15 and CCL19” −secreting CAR−T cells in human glioblastoma orthotopic xenograft model

**DOI:** 10.3389/fonc.2025.1539055

**Published:** 2025-03-19

**Authors:** Wanqiong Chen, Limian Hong, Shaomei Lin, Na Xian, Cailing Yan, Ningning Zhao, Yonglei Xiao, Wanting Liao, Yuxiang Huang, Mingzhu Chen

**Affiliations:** ^1^ School of Pharmacy, Quanzhou Medical College, Quanzhou, Fujian, China; ^2^ Department of Pharmacy, Quanzhou First Hospital Affiliated to Fujian Medical University, Quanzhou, Fujian, China; ^3^ Institute of Immunotherapy, Fujian Medical University, Fuzhou, Fujian, China; ^4^ Tcelltech Biological Science and Technology Inc., Fuzhou, Fujian, China; ^5^ Public Technology Service Center, Fujian Medical University, Fuzhou, Fujian, China; ^6^ Laboratory Animal Center, Fujian Medical University, Fuzhou, Fujian, China

**Keywords:** CAR-T cells, glioblastoma, IL-15, CCL19, cancer immunotherapy, EGFRv III

## Abstract

Despite the remarkable success of CAR-T cell therapy in hematologic malignancies, its progress in solid tumors has been slow. Overcoming challenges such as the recruitment and infiltration of CAR-T cells into the tumor site and the survival issues in the harsh tumor microenvironment are crucial for successful application in solid tumors. In this study, CAR-T cells were engineered to secrete both IL-15 and CCL19, and their efficacy was evaluated in a human glioblastoma orthotopic xenograft model. The results reveal that 15 × 19 CAR-T cells exhibit superior proliferation, chemotaxis, and phenotypic characteristics compared to conventional CAR-T cells *in vitro*. *In vivo*, 15 × 19 CAR-T cells exhibit superior control over tumors compared to conventional counterparts. Mechanistically, the improved efficacy can be attributed, in part, to IL-15 and CCL19 enhancing T-cell infiltration at the tumor site and fortifying resistance to exhaustion within the tumor microenvironment. In conclusion, the incorporation of IL-15 and CCL19 into CAR-T cells emerges as a promising strategy to elevate the anti-tumor efficacy of CAR-T cell therapy, positioning 15 × 19 CAR-T cells as a potential breakthrough for enhancing the application of CAR-T therapy in solid tumors.

## 1 Introduction

Glioblastoma (GBM) remains the most prevalent and aggressive form of brain tumor, with an annual incidence rate of approximately 3 per 100,000 (1). Despite employing diverse treatment modalities such as surgical resection, chemotherapy (typically temozolomide), radiotherapy and tumor-treating fields (TTF), the 5-year survival rate for GBM remains below 10% (2). Given the aggressive growth within the central nervous system (CNS), conventional treatments are often ineffective. The high malignancy, rapid progression, and high recurrence rate of GBM (3) necessitate the exploration of novel effective treatment approaches.

Immunotherapy has become a promising avenue for tumor treatment, offering more targeted advantages compared to traditional radiotherapy and chemotherapy. Chimeric antigen receptor T cell (CAR-T) therapy has demonstrated efficacy in hematological malignancies (4, 5). CAR-T cells are genetically engineered to express a single-chain variable fragment (scFv) specifically targeting tumor-associated antigens on the surface of T cells, enabling the elimination of tumor cells independently of major histocompatibility complex (MHC) recognition (6, 7). However, the success of CAR-T cell therapy in hematologic malignancies has not been replicated in solid tumors, with several challenges hampering the efficacy of CAR-T cell therapy in this context. Challenges include the limited T cell presence at the tumor site in certain solid tumors and the inefficiency of T cells to expand and maintain functionality in the hostile tumor microenvironment (TME) (8–10). While CAR-T cell studies for GBM treatment are underway, and some have progressed to clinical trials, the unique location, highly aggressive nature, and profoundly immunosuppressive TME of GBM present substantial challenges that clinical trials currently struggle to meet (11–14).

Previous studies have revealed the pivotal role of Interleukin-15 (IL-15), a growth factor, in inhibiting the apoptosis of T, B, and NK cells while fostering their survival (15).Its importance further encompasses the regulation of T cell development and homeostasis (16). Simultaneously, IL-15 actively engages in the genesis, maintenance, and reactivation of naive T cells, effector T cells, and memory T cells (17). In tandem, C-C motif chemokine ligand 19 (CCL19), a chemotactic factor, can recruit T cells and dendritic cells (DCs) to migrate and infiltrate tumor sites (18, 19). These studies have motivated our interest in engineering the class III variant of the epidermal growth factor receptor (EGFRvIII)-targeted CAR-T cells designed to secrete IL-15 and CCL19. This dual action aims to not only sustain the functionality of T cells but also to attract T cells and DCs to the tumor site, ultimately improving the therapeutic efficacy of CAR-T cells against GBM.

## 2 Material and methods

### 2.1 Mice and cell lines

EGFRvIII^+^ U87 MG cell line overexpressing EGFRvIII officially donated from Ludwig Institute, University of California. EGFRvIII^+^ U87 MG cells were transduced with lentiviral vector encoding the luciferase reporter gene (pLenti CMV puro Luc, Miaoling biology) to generate EGFRvIII^+^ U87 MG-Luc cell line. 293T cells were acquired from TaKara company. All cell lines were incubated in DMEM medium (CORNING) supplemented with 10%FBS (PAN). NCG mice were purchased from Jiangsu Gempharmatech Biotechnology Co. Ltd. (Jiangsu, China). Animal studies were conducted in accordance with the Experiment Animal Care Commission of Quanzhou Medical College and raised in specific pathogen-free conditions at the Quanzhou Medical College Institute Experimental Animal Center (Quanzhou, China).

### 2.2 Construction of CAR vectors and generation of CAR-T cells

CAR vector construction followed a previously established methodology (20). In brief, the EGFRvIII specific CAR was constructed by linking the EGFRvIII specific scFv to a CD8α hinge, CD8α transmembrane domain, an intracellular domain of 4-1BB, and CD3ζ in a sequential manner. To incorporate human cytokines IL-15 and CCL19 into the CAR expression, two P2A peptides were employed to facilitate the individual expression of each cytokine. For the production of lentivirus, 293T cells were transfected using a vector expressing CAR along with two lentiviral packaging plasmids (psPAX2 and pMD2.G, maintained by Tcelltech Biological Science and Technology Inc.) employing a calcium phosphate cell transfection kit from Beyotime. The lentiviral supernatant was collected at 48 and 72 h, followed by concentration through ultracentrifugation. Human peripheral blood mononuclear cells (PBMCs) sourced from healthy volunteers underwent activation using T Cell TransAct (Miltenyi Biotech) according to the manufacturer’s guidelines. After 24 h, the calculated amount of virus (multiplicity of infection = 10) was introduced. The T cells were subsequently washed once after a 16-h co-incubation period.

### 2.3 Flow cytometry

The cells were incubated with corresponding flow cytometry antibodies at 4 ° C for 40 min, followed by two washes in phosphate-buffered saline (PBS) before being run on FACS Verse (BD Biosciences). To assess the expression of EGFRvIII specific CARs on T cells, phycoerythrin(PE)-labeled human EGFRvIII protein (Acrobiosystems) was utilized. For the measurement of CAR-T cell proliferation, CAR-T cells and mitomycin-C-treated target cells (5 µg/µL) were co-cultured on a 6-cell culture plate. The absolute number of CAR^+^ T cells was determined using precision count beads (Biolegend) on days 3 and 5. Additionally, for the evaluation the proportion of central memory T cells (T_CM_) cells, effector memory T cells (T_EM_), naive T cells (T_N_), or effector T cells (T_eff_) were stained with allophycocyanin (APC)-labeled anti-CCR7 (BD Bioscience), fluorescein isothiocyanate (FITC)-labeled anti-CD45RA (Biolegend) and peridinin chlorophyll protein complex/cyanine5.5 (PerCP/Cy5.5)-labeled anti-CD45RO (Biolegend) monoclonal antibodies. To assess apoptosis of CAR-T cells on day 15, FITC-labeled anti-annexin V (BD Bioscience) and 7-amino-actinomycin D (7-AAD) antibodies (BD Bioscience) were employed. To detect the exhaustion of CAR-T cells, FITC-labeled anti-TIM-3 (Biolegend), PerCP/Cy5.5-labeled anti-PD1 (Biolegend) and APC-labeled anti-LAG-3 monoclonal antibodies (BD Bioscience) were used. Tumor-infiltrating T lymphocytes (TILs) were analyzed using Fixable Viability Dye 506 (eBioscience), PE/Cy7-labeled anti-human CD3 (Biolegend).

### 2.4 Cytokine release assays

Untransduced (here-after referred to as UTD) T cells and CAR-T cells were co-cultured with target tumor cells at an effector-to-target ratio of 1:1 for 24 h. The supernatant was harvested after centrifugation at 500 g for 10 min to test the release of cytokines. The concentrations of IL-15 and CCL19 were measured by ELISA kits (MultiSciences Biotech; NeobioSciences Biotech).

### 2.5 *In vitro* cytotoxicity assay

To study the cytotoxicity of CAR-T cells, EGFRvIII^+^ U87 MG cells were labeled with 0.5 µM CFSE (Invitrogen) and co-cultured with CAR-T cells and UTD T cells at varying effector-to-target ratio of 8:1, 4:1, 2:1 and 1:1. A certain amount of UTD T cells were added to EGFRvIII CAR-T cells before co-cultured to make it consistent with CAR^+^ population of 15 × 19 EGFRvIII CAR-T cells. The mixed effector and target cells were combined in capped FACS tubes with a total volume 200 µL and centrifuged at 200 g for 1 min after mixing. After 10 h of co-culture, 0.1 µg DAPI (Invitrogen) was added to each sample, and the cells were immediately analyzed by flow cytometry within 5 s. The % CAR-T cells and UTD T cells lysis were calculated as follows: [(CFSE^+^DAPI^+^ cells−spontaneous apoptosis)/total CFSE^+^ cells] ×100%.

### 2.6 Transwell migration assay

The transwell migration assay followed the protocol described previously (21). Briefly, CAR-T cells and UTD T cells were stimulated with 5 µg/µL mitomycin-C-treated EGFRvIII^+^ U87 MG cells for 24 h to generate the co-culture supernatant. The supernatant was then added to the lower chambers (600 µL), while the T cells (activated for 1 day) were placed in the upper chambers. After 6 and 12 h, the cells migrated from the upper chamber to the lower chamber and were counted using the cellometer auto T4 bright field cell counter (Nexcelom Bioscience). Furthermore, to evaluate the role of CCR7 in T cell migration, T cells were incubated with anti-CCR7 monoclonal antibodies (Biolegend) for 1 h at 37 ° C to block CCR7. The absolute number of migrated cells was then determined using the same methodology described above.

### 2.7 Tumor models and treatment

In the human GBM orthotopic xenograft model, 4 × 10^4^ EGFRvIII^+^ U87 MG-Luc cells were injected intracranially into 5- or 6-week-old NCG mice using an animal brain stereotaxic apparatus, with four mice per group. Briefly, the procedure involved locating the target position, using the anterior fontanel as a starting point, 0.50 mm anterior to the fontanel and 1.5 mm to the left of the lateral sagittal suture, and drilling a hole. The target position was confirmed again, and the needle was slowly inserted at a depth of 4.5 mm. The day of tumor cell injection was recorded as day 0. Mice were subjected to imaging using the IVIS imaging system (IVIS Spectrum, PerkinElmer) on day 7. Based on the *in vivo* imaging results, tumor-bearing mice were ranked by tumor size and randomly divided into three groups, each containing four mice. Group assignment was based on ensuring no significant difference in tumor size among the groups. A certain amount of UTD T cells were added to EGFRvIII CAR-T cells to make it consistent with CAR^+^ population of 15 × 19 EGFRvIII CAR-T cells. On day 8, 6 × 10^6^ CAR^+^ T cells and the corresponding amount UTD T cells were intravenously injected, respectively. Tumor size in each group of mice was measured by *in vivo* imaging on days 11, 16, 23, 30, and 37. Mouse body weight was measured every 5 days. On day 63, mice were sacrificed, and blood, spleen, and bone specimens were collected to prepare single-cell suspensions for flow cytometry analysis. For the analysis of tumor-infiltrating T lymphocytes (TILs), separate experiments were performed with the same treatments (n = 3). Mice in each group were sacrificed five days after CAR-T cell injection to analyze the function and number of TILs.

### 2.8 Statistical analysis

Statistical evaluation was conducted using GraphPad Prism software version 6.0. Data are shown of a minimum of three biological replicates or independent experiments. All data are presented as the means ± SD unless otherwise specified. *P* values < 0.05 were considered to be significant. *P< 0.05, **P<0.01, ***P<0.001****P<0.0001.

## 3 Results

### 3.1 Generation of anti−human EGFRvIII CAR−T cells secreting IL−15 and CCL19

**Figure 1:**
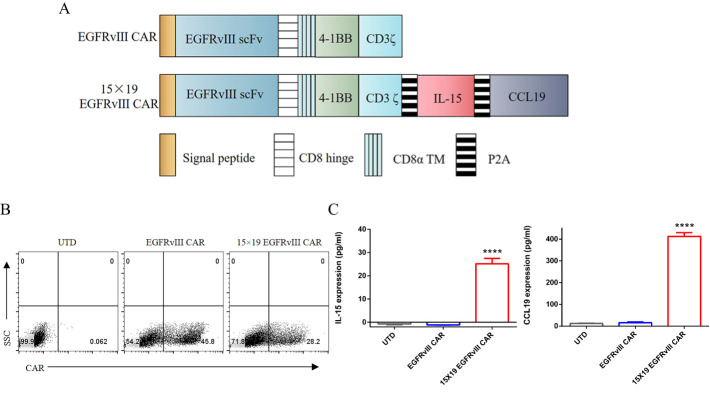
Generation of EGFRvIII specific CAR-T cells secreting IL-15 and CCL19. **(A)** Schematic representation of the lentiviral vector construction of EGFRvIII specific CAR secreting IL-15 and CCL19. **(B)** Flow cytometry analysis depicting the transduction efficiency of lentiviral vectors in transduced human T cells. The expression of EGFRvIII specific CAR was determined using the PE-labeled human EGFRvIII protein. **(C)** UTD T cells and CAR-T cells were co-cultured with target cells for 24 h. The supernatants from these co-cultures were collected for ELISA to measure the levels of IL-15 (left) and CCL19 (right). Each experiment was repeated independently 3 times, and representative data are shown. Data are the means ± SD of 3 independent experiments, ****P<0.0001.

We constructed anti-human EGFRvIII CAR incorporating signaling motifs consisting of 4-1BB and CD3ζ sequences, coupled with IL-15 and CCL19 sequences through a P2A oligopeptide (designated as 15 × 19 EGFRvIII CAR) (**Figure 1A**). Additionally, we constructed a conventional anti-human EGFRvIII CAR, serving as a control (referred to as EGFRvIII CAR). Upon lentiviral vector transduction of human PBMCs with either EGFRvIII CAR or 15 × 19 EGFRvIII CAR, transduction efficiencies were approximately 45% and 28%, respectively (**Figure 1B**). Notably, significant secretion of IL-15 (25.17 ± 2.3 pg/mL) and CCL19 (412.6 ± 17.2 pg/mL) was observed in the supernatant of co-cultures involving EGFRvIII^+^ U87 MG cells and 15 × 19 EGFRvIII CAR-T cells, contrasting with minimal secretion in co-cultures involving EGFRvIII CAR-T cells or untransduced (referred to as UTD) T cells (**Figure 1C**). *P*<0.0001, one-way ANOVA was performed to assess differences between groups.

### 3.2 15×19 EGFRvIII CAR-T cells showed better proliferation, migration and anti-apoptosis *in vitro*

**Figure 2:**
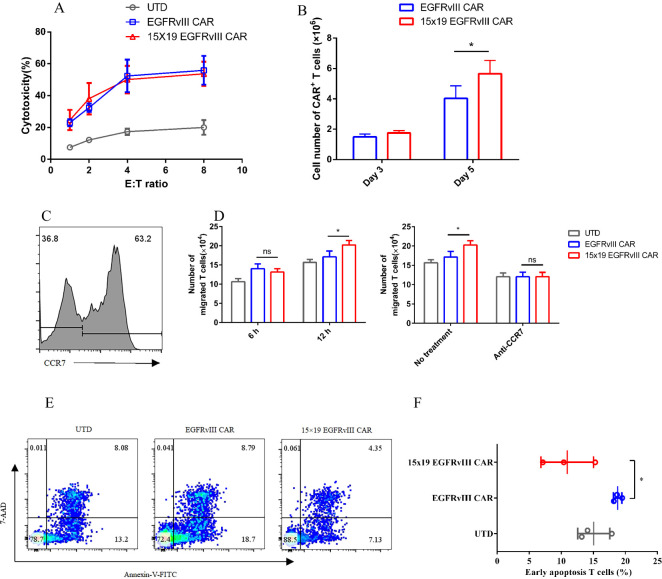
Enhancement of T cell T cells’ proliferation, migration, and anti-apoptosis by 15 × 19 EGFRvIII CAR T cells. **(A)** EGFRvIII CAR-T cells, 15 × 19 EGFRvIII CAR-T cells, or UTD T cells, were co-cultured with EGFRvIII^+^ U87 MG cells for 10 h. Subsequently, the cytotoxicity against target cells by UTD T cells and CAR-T cells was analyzed. **(B)** Co-cultures of EGFRvIII CAR-T cells and 15 × 19 EGFRvIII CAR-T cells with mitomycin C-treated EGFRvIII^+^ U87 MG cells were conducted. The absolute number of CAR^+^ T cells was measured using precision count beads via flow cytometry on days 3 and 5. **(C)** Human CCR7 expression on T cells was analyzed on day 1 post-activation using flow cytometry. **(D)** CAR-T cells were stimulated with mitomycin-C-treated EGFRvIII^+^ U87 MG cell for 24 h. The supernatant was then transferred to the lower chambers, while T cells were placed in the upper chambers. Migration of T cells to the lower chamber was quantified after 6 and 12 h **(E, F)**. The percentage of apoptotic T cells was detected by flow cytometry on day 15. Data represent the mean ± SD of triplicate independent experiments, *P < 0.05.

To assess cytotoxic activity, we compared 15 × 19 EGFRvIII CAR-T cells and EGFRvIII CAR-T cells in co-culture with target cells at varying effector-to-target (E:T) ratios. Remarkably, no significant difference in cytotoxic activity was observed between the two groups (**Figure 2A**). To elucidate the effect of IL-15 on the proliferation of CAR-T cells, we co-cultured EGFRvIII CAR-T cells and 15 × 19 EGFRvIII CAR-T cells with U87 MG cells expressing EGFRvIII that were treated with mitomycin C for five days. Notably, on day 5, the number of CAR^+^ T cells in the 15 × 19 EGFRvIII CAR group (5.89 ± 0.7 × 10^6^ cells) was significantly higher than that in the EGFRvIII CAR group (4.03 ± 0.8 × 10^6^ cells) (*P* < 0.05, two-tailed unpaired t-test) (**Figure 2B**). This represents a 46.2% increase in cell proliferation in the 15 × 19 EGFRvIII CAR group compared to the EGFRvIII CAR group. Furthermore, to assess the chemotactic function of CCL19 secreted by 15 × 19 EGFRvIII CAR-T cells, transwell migration assays were performed. T cells (one day after activation) expressing high levels of CCR7 were in the upper chamber (**Figure 2C**). Results demonstrated a greater migration of T cells to the lower chamber in the 15 × 19 EGFRvIII CAR group (20.25 ± 1.12 × 10^4^ cells) compared to both the UTD (15.67 ± 0.78 × 10^4^ cells) and EGFRvIII CAR groups (17.14 ± 1.49 × 10^4^ cells) (*P* < 0.05, one-way ANOVA followed by Turket’s multiple comparisons test) (**Figure 2D**, left). However, pre-incubation of T cells and CCR7 antibody for 1 h abolished these differences (**Figure 2D**, right). Given the role of IL-15 in preventing apoptosis and promoting T-cell survival, we evaluated apoptosis levels among different CAR-T cells. As expected, 15 × 19 EGFRvIII CAR-T cells (10.94 ± 4.1%) exhibited lower levels of apoptosis compared to EGFRvIII CAR-T (18.80 ± 0.7%) (*P* < 0.05, one-way ANOVA) (**Figures 2E, F**).

### 3.3 Phenotypic features of 15×19 EGFRvIII CAR-T cells

**Figure 3:**
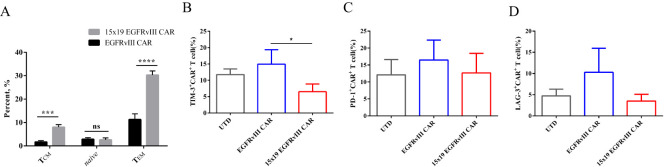
Phenotypic features of 15 × 19 CAR-T cells. **(A)** Flow cytometry analysis of CAR-T cells’ differentiation phenotype upon antigen stimulation, delineating subsets including naive (CD45RO^-^CCR7^+^), T_CM_ (CD45RO^+^CCR7^+^), and T_EM_ (CD45RO^+^ CCR7^-^) T cells. The expression levels of TIM-3 **(B)**, PD-1 **(C)**, and LAG-3 **(D)** were determined by flow cytometry. Data are presented as means ± SD from three independent experiments, and one-way ANOVA is used for comparison of phenotypic features of CAR-T cells. *P<0.05; ***P<0.001; ****P<0.0001; ns, not significant.

Given the pivotal role of T cell differentiation status in their persistence and anti-tumor efficacy (22), we detected the differentiation phenotype of CAR-T cells upon antigen stimulation (co-cultured with mitomycin C-treated EGFRvIII^+^ U87 MG cells). The findings revealed that a greater proportion of 15 × 19 EGFRvIII CAR-T cells exhibited the CD45RO^+^CCR7^+^ central memory T cells (T_CM_) phenotype compared to EGFRvIII CAR-T cells. The findings revealed that the proportion of CD45RO^+^CCR7^+^ central memory T cells (T_CM_) in 15 × 19 EGFRvIII CAR-T cells (8.03 ± 1.0%) was 4.7-fold higher than that in EGFRvIII CAR-T cells (1.70 ± 0.5%) (*P* < 0.001, two-tailed unpaired t-test) (**Figure 3A**). Additionally, we investigated the expression levels of inhibitory receptors, such as PD-1, TIM-3, and LAG-3, which are known to promote T-cell dysfunction and exhaustion upon activation (11). Flow cytometry analysis demonstrated that the expression of TIM-3 on 15 × 19 EGFRvIII CAR-T cells (6.50 ± 2.4%) was significantly lower than that on EGFRvIII CAR-T cells (14.90 ± 4.4%), *P* < 0.05 (**Figure 3B**). Although the expression levels of PD-1 and LAG-3 did not significantly differ between the two CAR-T cell types, 15 × 19 EGFRvIII CAR-T cells displayed a trend toward lower expression. Specifically, the expression of PD-1 was 12.68 ± 3.3% in 15 × 19 EGFRvIII CAR-T cells compared to 16.47 ± 3.3% in EGFRvIII CAR-T cells, while the expression of LAG-3 was 3.49 ± 0.9% in 15 × 19 EGFRvIII CAR-T cells compared to 10.28 ± 3.2% in EGFRvIII CAR-T cells (**Figures 3C, D**).

### 3.4 Enhanced tumor regression efficacy of 15 × 19 EGFRvIII CAR−T cells in human glioblastoma orthotopic xenograft model

**Figure 4:**
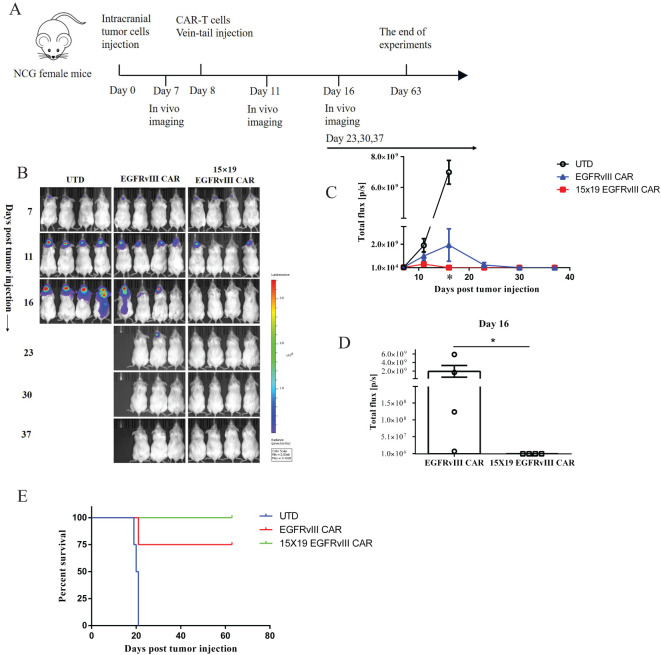
Anti-tumor effects of 15 × 19 EGFRvIII CAR-T cells in human GBM orthotopic xenograft models. **(A)** Schematic representation of the *in vivo* anti-tumor experiment. NCG mice were intracranially injected with EGFRvIII^+^ U87 MG-Luc cells and subsequently treated with intravenous injections (i.v) of CAR-T cells or UTD T cells (n = 4). **(B)** Assessment of tumor growth using the IVIS system at different time points. **(C)** Calculations of total flux (p/ s) using Living Image software at different time points. **(D)** Calculation of total flux (p/s) in the CAR-T cells group on day 16. Error bars denote SEM, *P<0.05. **(E)** The percentage survival per group was determined and is represented in a Kaplan–Meier survival curve.

We investigated the anti-tumor effects of 15 × 19 EGFRvIII CAR-T cells in an orthotopic model of human GBM. Immunodeficient NCG mice received intracranial injections of EGFRvIII^+^ U87 MG-Luc cells on day 0. Subsequently, CAR-T cells or UTD cells were administered via the tail vein on day 8. Tumor growth was monitored using *in vivo* imaging with the IVIS system (**Figure 4A**). Treatment with both EGFRvIII CAR-T and 15 × 19 EGFRvIII CAR-T cells effectively inhibited the growth of the intracranial tumors. Notably, the tumor regression efficacy of 15 × 19 EGFRvIII CAR-T cells significantly outperformed that of EGFRvIII CAR-T cells (tumor growth data were analyzed with two-way ANOVA) (**Figures 4B, C**). Specifically, treatment with 15 × 19 EGFRvIII CAR-T cells resulted in complete tumor regression in 100% of the mice on day 16 (8 days post-CAR-T cells), the mean bioluminescence signal was 3.38×10^5^ ± 25216 p/s, exhibiting rapid tumor eradication, while the mean bioluminescence signal of EGFRvIII CAR-T cells was 1.93 ± 1.4×10^9^ p/s (*P*< 0.05, two-tailed unpaired t-test) (**Figure 4D**). In contrast, among EGFRvIII CAR-treated mice, one out of four (25%) succumbed to the disease by day 20, and the remaining mice (3 of 4, 75%) did not achieve tumor regression until day 30, which was 14 days slower than observed with 15 × 19 EGFRvIII CAR-T cells. Notably, no significant reduction in mouse body weight was observed in the 15 × 19 EGFRvIII CAR-T cell-treated group (**Supplementary Figure S1**). Survival times were recorded for each group of mice (**Figure 4E**). Thus, our results underscored the promising therapeutic potential of 15× 19 EGFRvIII CAR-T cells in the orthotopic model of human GBM.

### 3.5 Mechanistic analyses of accelerated tumor regression of 15 × 19 EGFRvIII CAR−T cells in human glioblastoma orthotopic xenograft model

**Figure 5:**
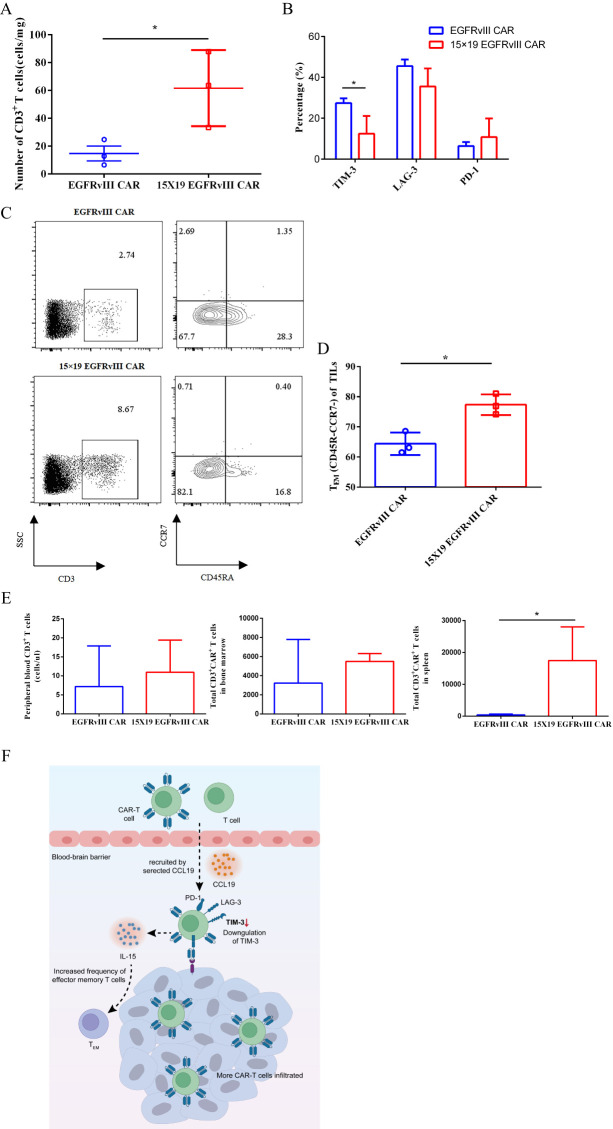
Mechanistic analyses of accelerated tumor regression of 15 × 19 EGFRvIII CAR−T cells in human GBM orthotopic xenograft model. NCG mice were intracranially implanted with EGFRvIII^+^ U87 MG-Luc cells on day 0, followed by i.v. injections of EGFRvIII CAR−T or 15 × 19 CAR-T cells on day 8. On day 13 (5 days post-CAR-T cell injection), mice were euthanized, and tumor masses were extracted to obtain single-cell suspensions for flow cytometric analysis. Expression levels of human CD3, EGFRvIII specific CAR, TIM-3, PD-1, and LAG-3 were examined. **(A)** The number of T cells at the tumor site following treatment with both types of CAR-T cells were shown. **(B)** The expression levels of TIM-3, PD-1, and LAG-3 of TILs were assessed. **(C)** Representative flow cytometric images depicting the percentage of T cells in tumor mass and the percentage of effector memory T cells (T_EM_, CD45RA^-^CCR7^-^) among T cells. **(D)** Histogram illustrating the percentage of T_EM_ cells within TILs. **(E)** On day 63 (at the end of experiments), mice were euthanized, and single-cell suspensions were prepared from blood, spleen, and bone specimens for flow cytometry analysis to determine the number of CAR-T cells *in vivo*. **(F)** The schematic provides a depiction of the proposed mechanisms through which 15 × 19 EGFRvIII CAR−T cells augment their anti-tumor efficacy within the TME. * *P*<0.05.

To explore the mechanisms underlying the accelerated tumor regression observed with 15 × 19 CAR−T cells, we conducted analyses on the number and phenotypes of TILs five days after the injection of CAR-T cells. Remarkably, the number of TILs retrieved from the mice treated with 15 × 19 EGFRvIII CAR-T cells (61.61 ± 15.7 cells/mg) was approximately four times higher than those from the mice treated with EGFRvIII CAR-T cells (14.76 ± 5.3 cells/mg) (**Figure 5A**). These findings indicate that 15 × 19 EGFRvIII CAR-T cells promote infiltration of T cells by secreting both IL-15 and CCL19. Furthermore, we observed a significant downregulation of TIM-3 expression in TILs from the 15 × 19 EGFRvIII CAR group, consistent with the *in vitro* findings (**Figure 5B**). Notably, there was a higher proportion of effector memory T cells (T_EM_) in the TILs of mice treated with 15 × 19 EGFRvIII CAR-T cells (77.37 ± 2.0%) compared to those treated with EGFRvIII CAR-T cells (64.4 ± 2.1%) (**Figures 5C, D**). Additionally, single-cell suspensions of spleen, bone marrow, and peripheral blood were prepared to evaluate the persistence of CAR-T cells on day 63, the end of the experiments. We observed that, compared with the EGFRvIII CAR group (419.2 ± 136.5 cells), the 15 × 19 EGFRvIII CAR group (17464 ± 6103 cells) exhibited significantly higher number of CD3^+^CAR^+^ T cells in the spleen, with a trend towards greater numbers in peripheral blood and bone marrow (**Figure 5E**). Hence, based on the current findings, we propose a schematic representation to depict the potential mechanisms through which IL-15 and CCL19 augment the functionality of CAR-T cells within the tumor microenvironment (**Figure 5F**). These results indicated that 15 × 19 CAR-T cells not only demonstrated superior anti-tumor activity but also exhibited enhanced persistence, both in terms of quantity and quality.

## 4 Discussion

Numerous studies have focused on augmenting CAR-T cells with IL-15, which has been shown to enhance CAR-T cells expansion and maintain a phenotype believed to potentiate their *in vivo* anti-tumor activity. This modification has also been associated with reduced expression levels of exhaustion-associated molecules by CAR-T cells within the tumor microenvironment (23–28). Additionally, the unique challenges posed by the blood-brain barrier in GBM necessitate innovative approaches for CAR-T cell delivery to tumor sites. Recognizing these challenges, we adopted a strategy of co-loading CAR-T cells with IL-15 and CCL19, expecting that this approach would facilitate the migration and infiltration of CAR-T cells into the tumor site while also supporting their sustained expansion and anti-tumor functionality within the TME. In this study, we engineered CAR-T cells directed against EGFRvIII to secret human IL-15 and CCL19 and assessed their anti-tumor efficacy against GBM in an orthotopic mouse model. The results demonstrated a significant enhancement in the anti-tumor efficacy of CAR-T cells armed with IL-15 and CCL19, characterized by more robust and rapid tumor control. Analysis of TILs indicated that the enhanced anti-tumor efficacy of 15 × 19 EGFRvIII CAR-T cells was partly attributable to their enhanced ability to infiltrate and/or proliferate within the tumor site. These cells exhibited a greater proportion of T_EM_ cells and demonstrated resistance to exhaustion within the TME.

The unfavorable immunosuppressive TME imposed constraints on T cell expansion, leading to dysfunction or anergy, a phenomenon also observed in CAR-T cells (29, 30). Previous studies have demonstrated that IL-15 can inhibit the apoptosis of T cells and improve T cell survival (15). Consistent with these findings, the results indicated that IL-15 promoted the proliferation of CAR-T cells and prevented the apoptosis of CAR-T cells *in vitro*. Additionally, we observed a greater presence of T cells in tumor tissue in the 15 × 19 EGFRvIII CAR-T cells group. IL-15 has also been shown to reduce the expression level of exhaustion-associated molecules, especially TIM-3 (23, 26). Our results corroborated these findings, revealing lower expression levels of exhaustion-associated molecules in 15 × 19 EGFRvIII CAR-T cells compared to EGFRvIII CAR-T cells, both *in vitro* and *in vivo*. Therefore, we hypothesized that the enhanced tumor control demonstrated by 15 × 19 EGFRvIII CAR-T cells may be due in part to the enhanced proliferation capacity and their resistance to exhaustion within the TME.

Furthermore, IL-15 plays an important role in the generation, maintenance, and reactivation of naive T cells, effector, and memory T cells (17). Therefore, we assessed the phenotype of CAR-T cells secreting IL-15 and CCL19 in response to antigen stimulation *in vitro*. Our results revealed that, following antigen stimulation, 15 × 19 EGFRvIII CAR-T cells displayed a greater proportion of T_CM_ cell subsets. The phenotype of CAR-T cells has been shown to be a critical determinant of clinical efficacy (22, 31, 32). Evidence suggests that infusion of less differentiated CAR-T cells leads to increased *in vivo* expansion, prolonged persistence, and enhanced anti-tumor efficacy (33–35). T_CM_, characterized by increased proliferative potential (36) and greater retention *in vivo*, are considered ideal candidates for CAR-T cell therapy (37). Therefore, we propose that the enhanced tumor control observed with 15 × 19 EGFRvIII CAR-T cells in the orthotopic xenograft model may be partly attributed to the introduction of IL-l5, resulting in a less differentiated and more functionally viable phenotype of CAR-T cells cultured *in vitro*. However, the absence of a rechallenge model restricts the capacity to establish conclusive evidence regarding this issue.

GBM is identified as “intracranial neoplasms,” presents a formidable challenge for systemically administered CAR-T cells. These cells must navigate through the blood circulation, traverse the blood-brain barrier, and penetrate the stiffen tumor-associated extracellular matrix (ECM) before encountering tumor cells (38). Previous studies have highlighted the role of CCL19 in promoting immune cell migration and infiltration to tumor sites (39, 40). Consistent with these findings, our observations demonstrated that CCL19 promoted T cell migration *in vitro*, and we observed an increased presence of TILs in the 15 × 19 EGFRvIII CAR-T cell-treated group in animal experiments. However, elucidating whether the observed increase in TILs was attributable to the migratory properties of CCL19 or the expansion-inducing effects of IL-15 warrants further investigation, possibly through real-time observation of CAR-T cell distribution experiments (41).

While a previous study introduced IL-15 and CCL19 into CAR-T cells and evaluated the anti-tumor effects using a zebrafish xenotransplantation model (42), mouse xenograft models remain the gold standard for preclinical evaluation of CAR-T cell therapy. Thus, to our knowledge, this study is the first to evaluate the therapeutic efficacy of IL-15 and CCL19-producing CAR-T cells using a mouse orthotopic xenotransplantation model. Our findings reveal the promising potency of 15 × 19 CAR-T cells for clinical application.

In summary, the study represents the first demonstration that CAR-T cells expressing human IL-15 and CCL19 can promote therapeutic efficacy against GBM using a human GBM orthotopic mouse xenograft model. While further exploration is needed to elucidate the detailed mechanisms underlying the enhanced anti-tumor efficacy of 15 × 19 CAR-T cells and to ensure their safety, our current results are encouraging and provide evidence for the clinical development of 15 × 19 EGFRvIII CAR-T cells for GBM and cancers positive for EGFRvIII expression.

## Data Availability

The original contributions presented in the study are included in the article/**Supplementary Material**. Further inquiries can be directed to the corresponding author.
