# Correction: Enhanced anti−tumor efficacy of “IL−15 and CCL19” −secreting CAR−T cells in human glioblastoma orthotopic xenograft model

**DOI:** 10.3389/fonc.2025.1645247

**Published:** 2025-08-13

**Authors:** Wanqiong Chen, Limian Hong, Shaomei Lin, Na Xian, Cailing Yan, Ningning Zhao, Yonglei Xiao, Wanting Liao, Yuxiang Huang, Mingzhu Chen

**Affiliations:** ^1^ School of Pharmacy, Quanzhou Medical College, Quanzhou, Fujian, China; ^2^ Department of Pharmacy, Quanzhou First Hospital Affiliated to Fujian Medical University, Quanzhou, Fujian, China; ^3^ Institute of Immunotherapy, Fujian Medical University, Fuzhou, Fujian, China; ^4^ Tcelltech Biological Science and Technology Inc., Fuzhou, Fujian, China; ^5^ Public Technology Service Center, Fujian Medical University, Fuzhou, Fujian, China; ^6^ Laboratory Animal Center, Fujian Medical University, Fuzhou, Fujian, China

**Keywords:** CAR-T cells, glioblastoma, IL-15, CCL19, cancer immunotherapy, EGFR vIII

In the published article, there was an error in **Figure 4B** as published. During figure compilation, the image intended to represent the first mouse in the UTD group was erroneously placed as the third mouse in the EGFRvIII CAR group on day 7 post-tumor-cell injection, resulting in image duplication in **Figure 4B**. This was an unintentional error in image placement. The corrected **Figure 4** and its caption appear below.

**Figure 4:**
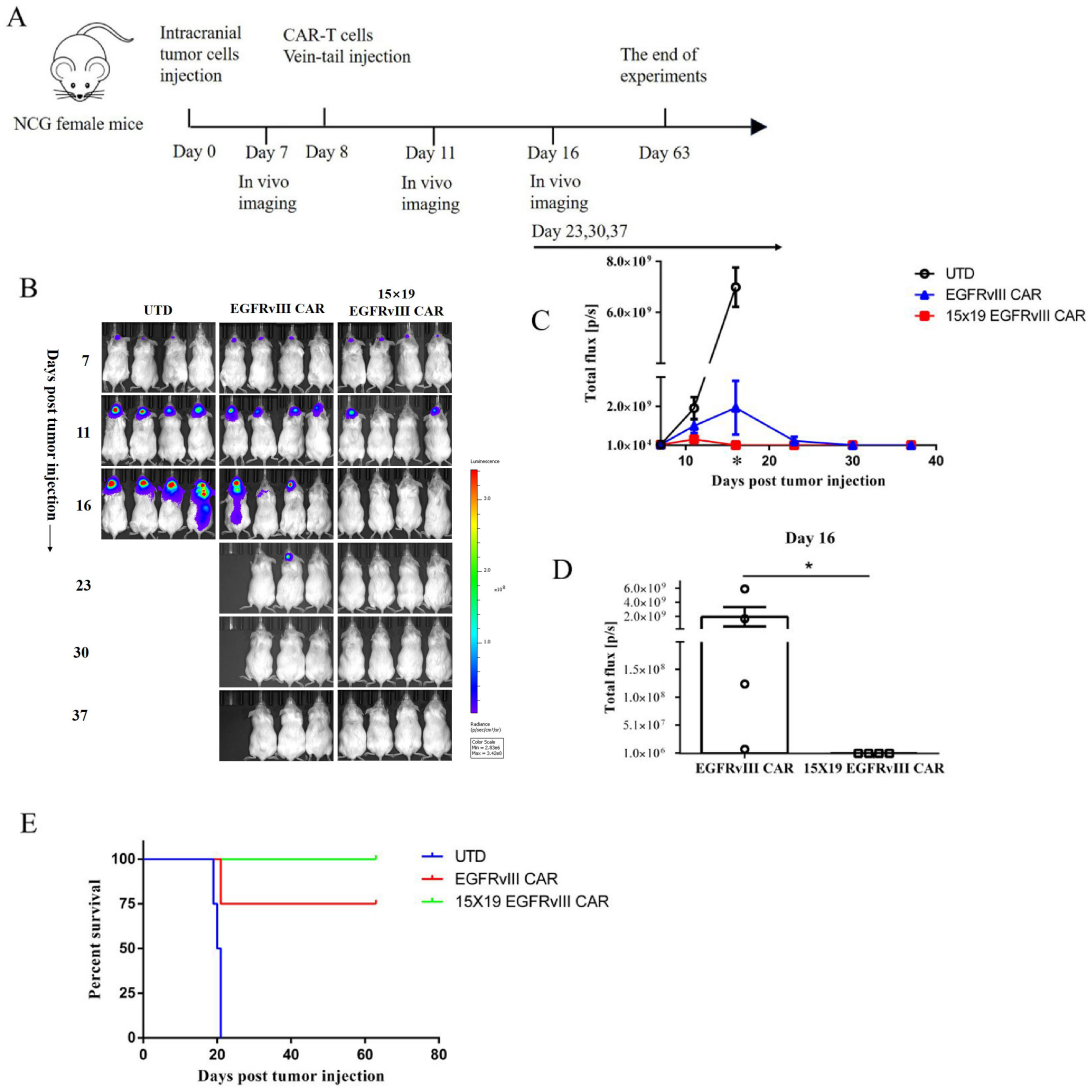
Anti-tumor effects of 15 × 19 EGFRvIII CAR-T cells in human GBM orthotopic xenograft models. **(A)** Schematic representation of the in vivo anti-tumor experiment. NCG mice were intracranially injected with EGFRvIII^+^ U87 MG-Luc cells and subsequently treated with intravenous injections (i.v) of CAR-T cells or UTD T cells (n = 4). **(B)** Assessment of tumor growth using the IVIS system at different time points. **(C)** Calculations of total flux (p/s) using Living Image software at different time points. **(D)** Calculation of total flux (p/s) in the CAR-T cells group on day 16. Error bars denote SEM, *P<0.05. **(E)** The percentage survival per group was determined and is represented in a Kaplan–Meier survival curve.

The original version of this article has been updated.

