# Supplementary material for: Enhanced anti−tumor efficacy of “IL−15 and CCL19” −secreting CAR−T cells in human glioblastoma orthotopic xenograft model
Source: Front Oncol. 2025 Mar 19;15:1539055. doi: 10.3389/fonc.2025.1539055 (PMC11962218; doi:10.3389/fonc.2025.1539055)
Supplement: Supplementary file 1 [file DataSheet1.pdf]

Supplementary Material

Enhanced anti-tumor efficacy of "IL-15 and CCL19" -secreting CAR-T cells in human glioblastoma orthotopic xenograft model

Wanqiong Chen[1†], Limian Hong[2†], Shaomei Lin[1†], Na Xian[3, 4], Cailing Yan[5], Ningning Zhao[6], Yonglei Xiao[3], Wanting Liao[1], Yuxiang Huang[1], Mingzhu Chen[1*]

*** Correspondence:** Mingzhu Chen: 2008025@qzmc.edu.cn

1.1 Supplementary Figures

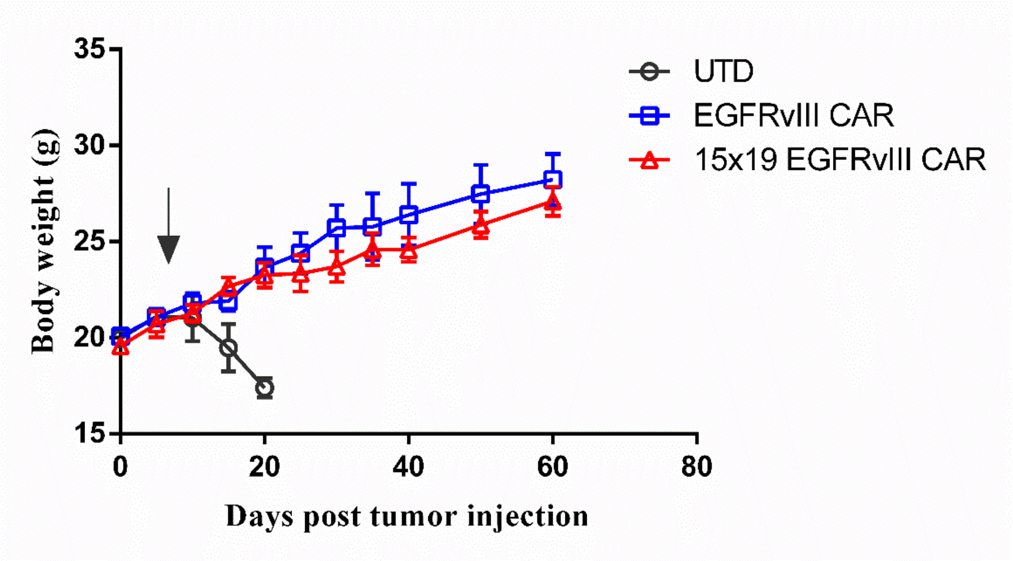

Supplementary Figure 1. Mouse body weight was measured every 5 days. Error bars denote SEM. The arrows in the figure indicate the time of CAR-T cells injection.
